# Evaluation of GPA, molGPA and prognostic factors in melanoma brain metastases: a single-center study

**DOI:** 10.1007/s12094-025-04118-2

**Published:** 2025-12-03

**Authors:** Jens Christian Philippi, Niklas Mittenbacher, Dirk Vordermark, Daniel Medenwald, Jörg Andreas Müller

**Affiliations:** 1 Department of Radiation Oncology, University Hospital Halle (Saale), Ernst-Grube-Str. 40, 06120 Halle (Saale), Germany; 2 University Clinic for Radiation Therapy, University Hospital Magdeburg A. ö. R, Leipziger Str. 44 39120, Magdeburg, Germany

**Keywords:** Melanoma brain metastases, Graded prognostic assessment (GPA), Molecular GPA (molGPA), Prognostic factors, Radiotherapy, Survival analysis

## Abstract

**Background:**

Malignant melanoma ranks among the leading causes of brain metastases (BM). The Graded Prognostic Assessment (GPA) and the melanoma-specific molecular GPA (molGPA) are well-established prognostic tools that help estimate survival and guide therapeutic decisions. This single-center, retrospective clinical study aimed to evaluate prognostic factors for overall survival (OS), intracranial tumor control (IC), and intracranial progression-free survival (IPFS), as well as GPA and molGPA within a real-world clinical setting.

**Methods:**

Patients with melanoma-associated BM who underwent radiotherapy (RT) between January 2016 and December 2023 were included. Study endpoints included OS, IC, and IPFS, with OS and IPFS analyzed across the entire cohort (*n* = 59) and IC was evaluated in cases with follow-up imaging (*n* = 47).

**Results:**

The median survival of all patients was 6.9 months (IQR 2.2; 23.6). Regarding OS, three predictive factors were significantly associated with the survival probability: a Karnofsky performance status index (KPS) <70 (*p* = 0.003), a GPA score of 0–1.0 versus 1.5–4.0 (*p* = 0.002) and a molGPA score of 0–1.0 versus 1.5–4.0 (*p*= 0.034). Intracranial progression-free survival was significantly decreased in patients with KPS <70 (*p* = 0.01), multiple brain metastases (*p* = 0.036), and GPA scores of 0–1.0 (*p* = 0.011).

**Conclusions:**

Both GPA and molGPA were validated as significant predictors of OS in patients with melanoma-associated BM. However, GPA emerged as the sole score significantly associated with both OS and IPFS, suggesting a potential advantage in its clinical utility. The validation of GPA and molGPA as significant predictors for OS in a real clinical setting support their use in guiding treatment recommendations.

## Introduction

Malignant melanoma is the sixth most common cancer in Germany [1]. In 2020, approximately 23,560 people were diagnosed with malignant melanoma of the skin, two-third of these cases were detected at an early tumor stage (UICC I).

Furthermore, malignant melanoma is one of the most common causes of brain metastases (BM) with reported clinical incidences of 10–40%. The incidence of primary cutaneous melanoma has increased for several decades and remains the most lethal form of cutaneous neoplasm [2–4].

The median survival of patients diagnosed with melanoma BM is typically less than 1 year [3, 5–8]. Prognostic factors in terms of survival for patients with brain metastases from various tumor types include patient age, Karnofsky Performance Status (KPS), the presence of extracranial metastases, and the number of brain metastases. These variables are integrated into the Graded Prognostic Assessment (GPA) score, which provides a more refined estimation of survival and guides clinical decision-making for individualized treatment strategies [9].

Moreover, the melanoma-specific molecular GPA (molGPA), was developed to incorporate the BRAF mutation status as an additional prognostic factor by demonstrating the statistical significance of all included factors, this revision improved both the prognostic accuracy and clinical relevance of the score [10].

According to German and European guideline recommendations, treatment strategies for brain metastases include stereotactic radiosurgery or hypofractionated stereotactic radiotherapy or surgical intervention (with postoperative stereotactic radiotherapy) for patients with a limited number of metastases. Whole-brain radiotherapy (WBRT) is recommended for patients in whom local therapies have failed, those presenting with neurological symptoms and the need of corticosteroid treatment, or those with widespread metastatic disease. Nevertheless, the prognosis for this population is extremely poor and it is recommended to discuss and prepare palliative care [11, 12]. While WBRT is commonly advised for patients with multiple brain metastases, most studies could not demonstrate a statistically significant improvement in overall survival (OS) while one study showed benefits just for cerebral progression [7, 13–16]. In contrast, localized treatments such as surgery or stereotactic fractionated radiotherapy have shown a statistically significant survival benefit in patients with limited brain metastases [7, 15, 17].

Additionally, the use of monoclonal antibodies such as ipilimumab, nivolumab, pembrolizumab or combinations thereof is recommended. For patients with a confirmed BRAF mutation, BRAF inhibitors such as vemurafenib, dabrafenib, encorafenib or belvarafenib are viable treatment options. Both therapeutic approaches have been shown to significantly prolong OS in patients, often combined with radiotherapy (RT) [11, 15, 18].

The objective of this single-center retrospective clinical study was to identify potential prognostic factors for OS, intracranial control (IC) and intracranial progression-free survival (IPFS), as well as to evaluate the GPA and Melanoma molGPA within a real-world clinical setting.

## Methods

### Data and material

All patients with malignant melanoma-associated brain metastases treated in the Department of Radiation Oncology or the Department of Dermatology at Martin Luther University Halle-Wittenberg between January 2016 and December 2023 and initiated treatment with any form of radiotherapy (RT) were included in this study. The observation period extended until April 26, 2024, when we obtained the survival status for each patient through local citizen registration offices.

The study was approved by the ethics committee of the Medical Faculty of Martin Luther University Halle-Wittenberg (2025-007). Sociodemographic and clinical patient data were extracted from medical records using the hospital information system ORBIS version 03.20.02.01. Intracranial disease progression was evaluated through CT or MRI imaging using Centricity PACS by GE Healthcare Integrated IT Solutions Inc. and treatment plans were documented with Elekta Mosaiq version 2.84.

Endpoints were defined as overall survival (OS = defined as time period from the last day of RT until death or last Follow Up), intracranial tumor control (IC = absence of recurrence of treated metastases, absence of new metastases, absence of tumor progression of present brain metastases) and intracranial progression-free survival (IPFS = absence of recurrence of treated metastases, absence of new metastases, absence of tumor progression of present brain metastases, including death).

OS and IPFS were evaluated for the entire patient cohort (*n* = 59) and IC in *n* = 47 cases with any follow-up imaging.

Survival curves were generated using the Kaplan–Meier method to illustrate cumulative patient survival. Comparisons between groups were made using the log-rank test to evaluate statistical differences in survival times. To assess the impact of melanoma-specific GPA factors on survival, a Cox regression analysis was performed. At first, univariate regression models were used to evaluate potential prognostic factors. Then, all significant factors were assessed in a multivariate analysis. Statistical significance was accepted with two-sided *p*-values <0.05.

## Results

### Patient characteristics (Table 1)

**Table 1:** Patient demographics and clinical baseline characteristics

Overall		
(N = 59)		
Age		
Median		64.0
IQR		21.5
Gender		
Male		29 (49.2%)
Female		30 (50.8%)
BRAF Mutation V600E		
Mutation		25 (42.4%)
No mutation		34 (57.6%)
Karnofsky Performance Status Scale		
90		15 (25.4%)
80		13 (22.0%)
70		11 (18.6%)
60		18 (30.5%)
50		2 (3.4%)
Number of metastases at RT		
1		23 (39.0%)
2–4		20 (33.9%)
>4		16 (27.1%)
ECM		
Yes		50 (84.7%)
No		9 (15.3%)
GPA		
0–1.0		23 (39.0%)
1.5–2.0.5.0		28 (47.5%)
2.5–3.0.5.0		7 (11.9%)
Overall		
3.5–4.0.5.0	1 (1.7%)	
Melanoma molGPA		
0–1.0.0	21 (35.6%)	
1.5–2.0.5.0	26 (44.1%)	
2.5–3.0.5.0	11 (18.6%)	
3.5–4.0.5.0	1 (1.7%)	

The cohort consisted of 59 patients with a median age of 64 years (IQR 53.5; 75.0). 49.2% of patients were male (*n* = 29) and 50.8% were female (*n* = 30), resulting in an almost equal gender distribution. Molecular analysis revealed that 42.4% of patients (*n* = 25) carried a BRAF V600E mutation, whereas 57.6% (*n* = 34) did not. Regarding the clinical performance status, 15 patients (25.4%) presented with a KPS of 90, 11 patients (18.6%) exhibited a KPS of 70, and 20 patients (33.9%) were in poor clinical condition with a KPS of 60 or lower.

Extracranial metastases (ECM) were present in 84.7% (*n* = 50) of all cases. The cohort's median GPA score was skewed towards lower ranges, with 39.0% (*n* = 23) in the 0–1.0 range, 47.5% (*n* = 28) in the 1.5–2.0 range, and only one patient (1.7%) in the 3.5–4.0 range. Analogous, the melanoma-specific molecular GPA (molGPA) showed that 35.6% of patients (*n* = 21) scored 0–1.0, 44.1% (*n* = 26) scored 1.5–2.0, and only one patient (1.7%) had a score in the highest range of 3.5–4.0.

## Treatment (Table 2)

**Table 2:** Treatment characteristics of the study cohort

Overall	
(N = 59)	
Additional immune therapy or targeted therapy	
No	2 (3.4%)
Yes	57 (96.6%)
Immune and BRAF therapy agent	
Vemurafenib	4 (6.8%)
Dabrafenib	22 (37.3%)
Encorafenib	4 (6.8%)
Belvarafenib	1 (1.7%)
Pembrolizumab	25 (42.4%)
Nivolumab alone	25 (42.4%)
Ipilimumab alone	2 (3.4%)
Nivolumab & Ipilimumab	39 (66.1%)
Additional Chemotherapy	
Yes	6 (10.2%)
No	53 (89.8%)
Chemotherapy agent	
DTIC	4 (6.8%)
Temozolomid	1 (1.7%)
Gemcitabin & Treosulfan	1 (1.7%)
Radiotherapy	
WBRT	11 (18.6%)
Stereotactic Radiotherapy	38 (64.4%)
Both	10 (16.9%)

Radiotherapy modalities applied in the cohort included WBRT in 18.6% of patients (*n* = 11), stereotactic radiotherapy in 64.4% (*n* = 38), and a sequential combination of both approaches in 16.9% (*n* = 10). Almost all patients (96.6%, *n* = 57) received additional immune or targeted therapies. Only two patients (3.4%) were not treated with these agents. The most commonly used agents were dabrafenib (37.3%, *n* = 22), pembrolizumab (42.4%, *n* = 25), and nivolumab alone (42.4%, *n* = 25), with a larger proportion of patients (66.1%, *n* = 39) receiving a combination of nivolumab and ipilimumab. Additional chemotherapy was administered to 6 patients (10.2%) of the cohort, with agents such as DTIC (6.8%, *n* = 4), temozolomide (1.7%, *n* = 1), and gemcitabine combined with treosulfan (1.7%, *n* = 1).

Regarding metastatic burden at the time of RT, 39.0% of patients (*n* = 23) were diagnosed with a single metastasis, 33.9% (*n* = 20) had 2–4 metastases, and 27.1% (*n* = 16) had more than four metastases.

## Survival analyses (Tables 3, 4)

**Table 3:** Survival and progression outcome analyses

Overall	
(N = 59)	
Time from primary diagnosis to brain metastasis [months]	
Median	34.8
IQR	46.1
Missing	13 (22.0%)
Overall Survival [months]	
Median	6.90
IQR	21.4
Intracranial Progression	
Yes	33 (55.9%)
No	14 (23.7%)
Missing	12 (20.3%)
Intracranial Control	
Median	4.33
IQR	12.7
Missing	12 (20.3%)
Intracranial Progression-free Survival	
Median	3.43
IQR	7.18

**Table 4:** Univariable and multivariable cox regression analyses for survival and progression

	Overall survival UVA				Overall survival MVA				Intracranial progression-free survival UVA				Intracranial progression-free survival MVA			
	UVA				MVA				UVA				MVA			
	HR	95% CI Lower	95% CI Upper	p-value	HR	95% CI Lower	95% CI Upper	p-value	HR	95% CI Lower	95% CI Upper	p-value	HR	95% CI Lower	95% CI Upper	p-value
Age Group(<70 vs. > = 70)	1.641	0.886	3.038	0.115					1.118	0.634	1.973	0.700				
KPS (> = 70 vs. <70)	2.613	1.401	4.876	0.003	1.910	0.946	3.854	0.071	2.194	1.206	3.989	0.010	1.79	0.900	3.597	0.096
BRAF mutation (yes vs. no)	0.882	0.483	1.609	0.681					1.183	0.664	2.107	0.568				
Number of all brain mets (1 vs. >1)	1.824	0.964	3.452	0.065					1.865	1.042	3.336	0.036	1.612	0.862	3.014	0.135
Extra-cranial metastases (no vs. yes)	0.842	0.374	1.893	0.677					0.750	0.350	1.605	0.458				
GPA (1.5-4.0 vs. 0-1.0)	2.733	1.462	5.109	0.002	1.865	0.872	3.988	0.108	2.122	1.190	3.783	0.011	1.339	0.656	2.734	0.423
molGPA (1.5–4.0.5.0 vs. 0–1.0.0)	1.944	1.053	3.589	0.034	1.378	0.694	2.738	0.360	1.472	0.829	2.613	0.187				

The median time from primary diagnosis to brain metastasis was 34.8 months (IQR 16.4; 62.5). These data were missing for n = 13 patients (22%) due to an unknown primary tumor location. The median survival of all patients was 6.9 months (IQR 2.2; 23.6). On April 26^th^, 2024, when we obtained the survival status for each patient through local citizen registration offices, 15 patients (24,4%) were still alive while 44 (74,6%) had already died.

Regarding OS, three predictive factors were significantly associated with the survival probability: patients with a KPS < 70 had a hazard ratio (HR) of 2.61 (CI 1.40–4.88, *p* = 0.003) compared to those with a KPS ≥ 70. Likewise, patients with low GPA scores of 0–1.0.0 had a HR of 2.73 (CI 1.46–5.11, *p* = 0.002) related to patients with a GPA of 1.5–4.0, and patients with a molGPA of 0–1.0 demonstrated an HR of 1.94 (CI 1.05–3.59, *p* = 0.034) compared to those with a molGPA of 1.5–4.0.

No significant association with OS was observed for younger patients compared to patients older than 70 years (<70 vs. ≥70, HR = 1.64, CI 0.89–3.04), a positive BRAF mutation status in comparison to negative BRAF expression (HR = 0.88, CI 0.48–1.61), as well as the number of BM (1 vs. >1, HR = 1.82, CI 0.96–3.45), or the ECM status (HR = 0.84, CI 0.37–1.89).

The predictive factors KPS, GPA and molGPA which were significantly associated with OS in the univariate analysis were included in a multivariate model where none of these factors reached statistical significance (Table 4).

**Fig. 1:**
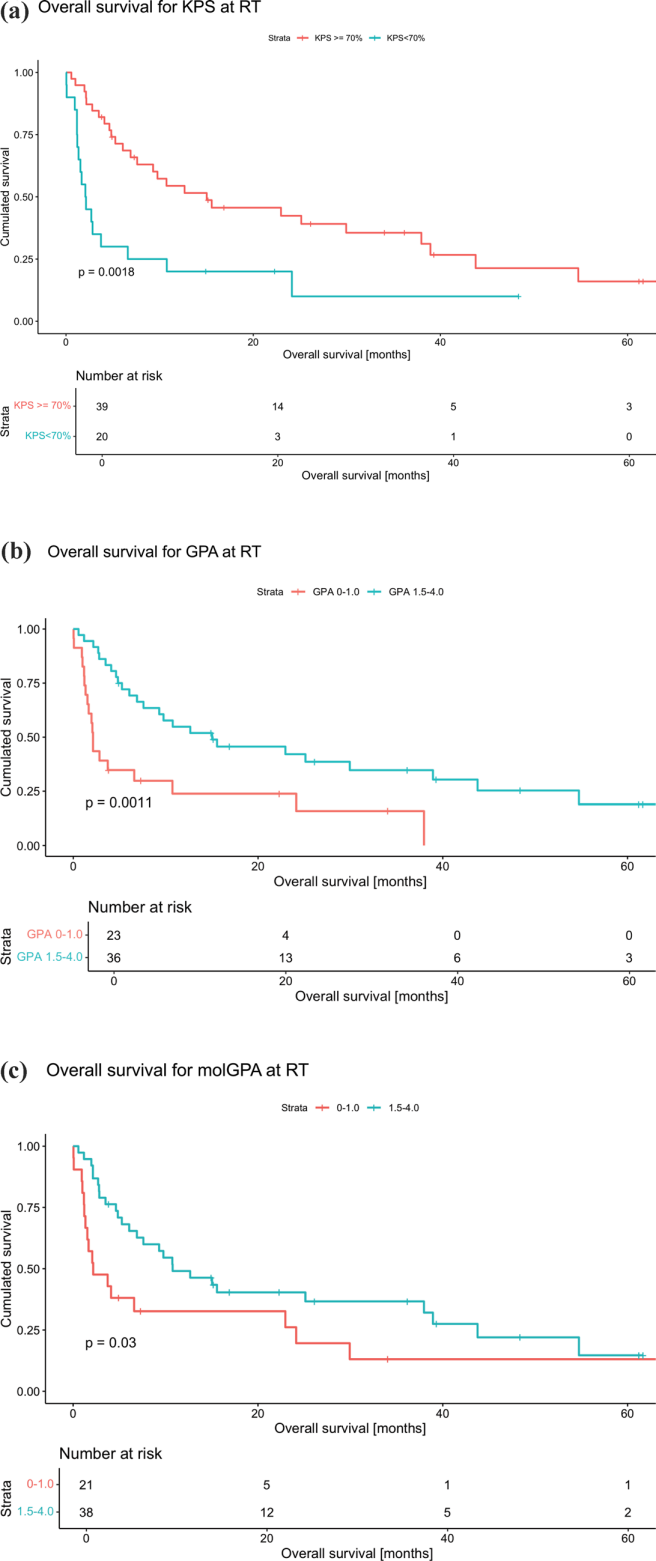
**A** Overall survival for KPS at RT. **B** Overall survival for GPA at RT. **C** Overall survival for molGPA at RT

Given their prior demonstrated significance, these variables were further analyzed in terms of cumulative survival over time following RT. Patients with a KPS ≥ 70 showed significantly improved median OS (15.0 months, *n* = 39) compared to those with a KPS < 70 (2.1 months, *n* = 20, *p* = 0.0018) (Fig. 1A). Patients with a GPA of 1.5–4.0 achieved a median OS (15.0 months, *n* = 36) while patients with a GPA of 0–1.0.0 exhibited a substantially lower median survival rate (2.1 months, *n* = 23, *p* = 0.0011) (Fig. 1B). Furthermore, MolGPA scores were significantly related to OS, with a median survival of 10.8 months (*n* = 38) for patients with a score of 1.5–4.0 compared to a median survival of 2.2 months (*n* = 21, *p* = 0.03) for patients with a score ranging from 0 to 1.0 (Fig. 1C).

Intracranial progression occurred in 55.9% (*n* = 33) cases, while 23.7% (*n* = 14) showed no signs of progression in their last imaging; due to the lack of imaging after RT, data on progression were missing for 20.3% (*n* = 12) of patients. The median duration of IC was 4.3 months (IQR 1.6; 14.3), while IPFS across the cohort was 3.4 months (IQR 1.3; 8.5).

Regarding IPFS, three predictive factors were again associated with a significantly increased HR. Patients with a KPS < 70 had an HR of 2.19 (CI 1.21–3.99, *p* = 0.01) compared to those with a KPS ≥ 70. Likewise, patients with more than one BM had an HR of 1.87 (CI 1.04–3.34, *p* = 0.036) relative to those with a single BM, and patients with a GPA score ranging from 0 to 1.0 showed an HR of 2.12 (CI 1.19–3.78, *p* = 0.011) compared to those with a GPA of 1.5–4.0.

No significant association was observed between IPFS and different age groups (<70 vs. ≥70, HR = 1.12, CI 0.63–1.97), BRAF mutation status (HR = 1.18, CI 0.66–2.11), ECM status (HR = 0.75, CI 0.35–1.61) or molGPA (0–1.0.0 vs. 1.5–4.0.5.0, HR = 1.47, CI 0.83–2.61).

The predictive factors KPS, number of BM and GPA which were significantly associated with IPFS in the univariate analysis have been included in a multivariate regression model where no significant association with IPFS could be shown. (Table 4)

**Fig. 2:**
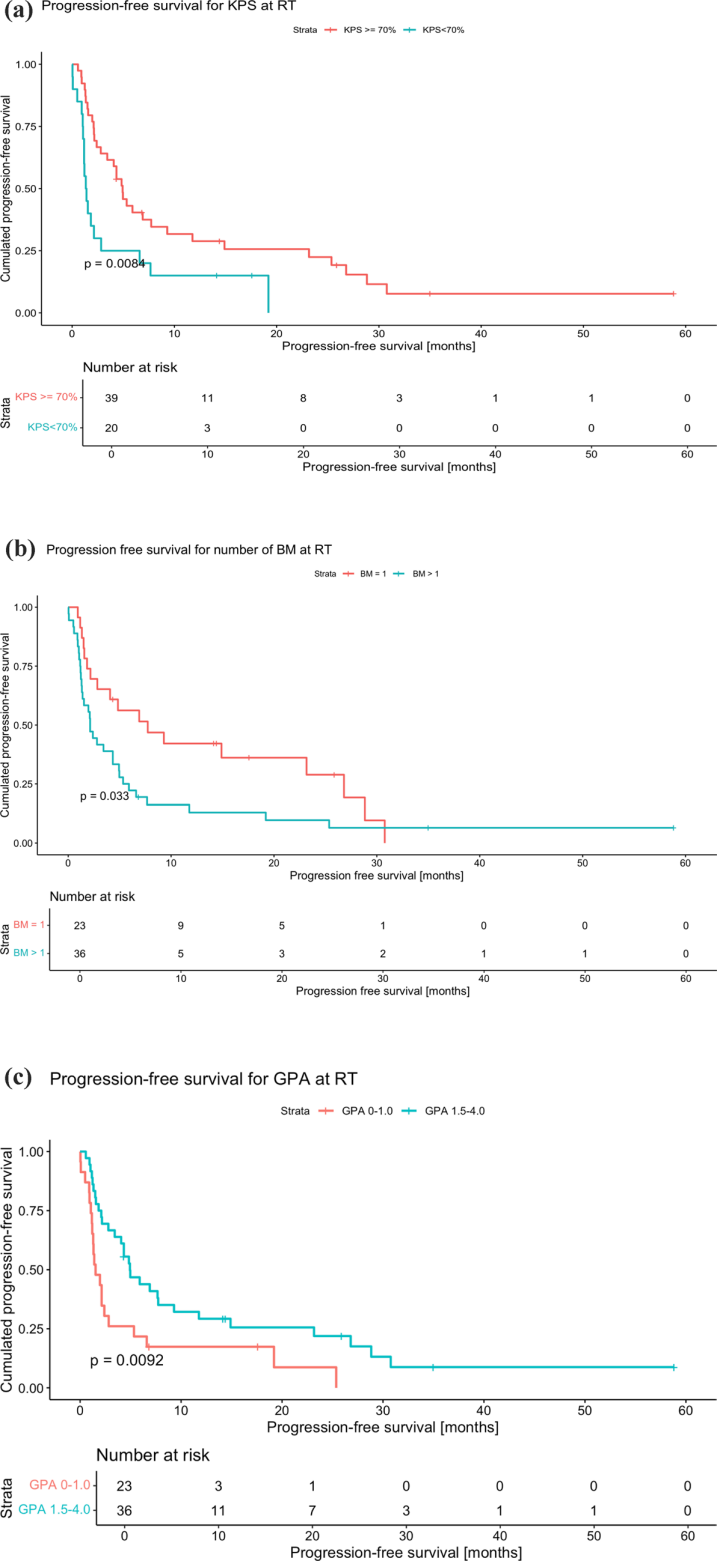
**A** Progression-free survival for KPS at RT. **B** Progression free survival for number of BM at RT. **C** Progression-free survival for GPA at RT

For the factors previously identified as significant, further analysis of cumulative IPFS over time following RT yielded the following results. Patients with a KPS ≥ 70 demonstrated superior IPFS (4.9 months, *n* = 39) compared to those with a KPS < 70 (1.4 months, *n* = 20, *p* = 0.0084) (Fig. 2A). The patient cohort with just one BM at RT showed a significant increase in IPFS. (7.7 months, *n* = 23) relative to the patient cohort with more than one BM at RT (2.1 months, *n* = 36, *p* = 0.033) (Fig. 2B). Patients with a GPA of 1.5–3.0 exhibited a higher IPFS (4.9 months, *n* = 36) compared to patients with a GPA of 0–1. (1.5 months, *n* = 23, *p* = 0.0092) (Fig. 2C).

For IC no significant associations were observed concerning any of aforementioned factors.

## Discussion

The objective of this study was the evaluation of the established prognostic scores GPA and molGPA for melanoma patients diagnosed with BM in a real-world clinical setting, as well as to identify outcomes and potential prognostic factors in these patients. Similar to previous studies [14, 17, 19], our study was retrospective, further emphasizing the need to confirm these findings in prospective, randomized trials.

Median OS was 6.9 months, and IPFS was 3.4 months. GPA had a significant impact on both OS (*p* = 0.002) and IPFS (*p* = 0.011), while molGPA significantly impacted OS (*p* = 0.034) but not IPFS.

Additional prognostic predictors with a significant association with the outcomes included a KPS ≥ 70 for OS and the presence of more than one BM for IPFS.

Previous studies identified age, KPS, LDH levels, BM count, leptomeningeal spread, presence of ECM, histology, neurological symptoms, and BRAF status as prognostic factors [10, 17, 19–21]. The median OS of 6.9 months in our study aligns with comparable studies in this field, such as Yu et al. (2002), Raizer et al. (2008), and Sperduto et al. (2017) [10, 19, 22]. Although median age at RT was slightly higher at 64 years, the median time from primary melanoma diagnosis to diagnosis of BM was similar to Kessel et al. (2021), Ostheimer et al. (2015) or Fife et al. (2004) with 34.8 months [7, 19, 23]. KPS and GPA scores in our cohort tended towards lower values related to these studies, while molGPA showed comparable results. Additionally, a higher proportion of patients in our study (39.0%) had only one BM [10, 19, 22].

The validation of GPA (*p* = 0.002) and molGPA (*p* = 0.034) as significant predictors for OS in a real-world clinical setting supports their use in guiding treatment recommendations. Survival estimates derived from GPA and molGPA provide clearer guidance for treatment decisions, supporting more aggressive approaches when higher GPA or molGPA scores indicate improved survival prospects. Conversely, when estimated survival is lower with a low GPA or molGPA, treatment decisions could be shifted towards best supportive care, such as hospice, to optimize patient quality of life [10].

While we were able to validate each score individually as a predictor for OS in patients with melanoma-associated BM, we could not demonstrate that molGPA is superior to GPA, as proposed by Sperduto et al. (2017) [10]. Instead, p-values favored GPA, which was also the only score shown to be statistically significant for both OS and IPFS. This may suggest that the inclusion of molecular markers in the molGPA does not necessarily improve prediction of intracranial disease control. One possible explanation could be the limited intracranial activity of systemic therapies such as BRAF/MEK inhibitors, which highlights the continued importance of combining local with systemic treatment approaches as discussed by Liao et al. [24]: however, the mentioned limitations of this study must be considered when interpreting these observations.

The lack of significant findings for other prognostic predictors such as age, ECM, or BRAF, as well as the absence of significance for molGPA in predicting IPFS, could potentially be attributed to the limited patient cohort in our study (*n* = 59) or perhaps to the generally poorer health status of these patients, as indicated by a trend toward lower GPA scores compared to similar studies [10, 19, 22]. In addition, this study is retrospective, introducing the inherent selection bias common to all retrospective analyses.

We identified only one predictor (KPS) within the GPA and molGPA scores as significant for OS, differing from the findings of Sperduto et al. [10]. This discrepancy could prompt questions about whether the scores' significance primarily derives from the strong predictive power of KPS, which may also reflect a somewhat subjective assessment of a patient’s health status. However, this concern should be set aside, as GPA showed even stronger significance with a lower p-value (0.002 vs. 0.003). The results may also be influenced by the limited sample size, with some predictors, such as the number of BM, closely missing significance. Also, these two factors, KPS as well as number of BM were the only two factors with significance in the first melanoma-GPA [10]. An inherent strength of both GPA and molGPA is their greater objectivity compared to similar scores.

In the most recent update of the melanoma GPA by Sperduto et al. [25], age and BRAF mutation status—both of which were also non-significant in our cohort—were excluded from the model. In contrast, newly identified prognostic factors such as elevated serum lactate dehydrogenase levels and the absence of prior immunotherapy before the development of melanoma brain metastases were incorporated to enhance the score’s prognostic accuracy.

## Conclusions

In summary, this study successfully evaluated both GPA and molGPA within a real-world clinical setting at a German university hospital and showed significant benefits. Although a slight statistical advantage was observed for GPA over molGPA, the study’s limitations prevent definitive conclusions about the superiority of either score, particularly considering their different levels of objectivity. In conclusion, both scores are significant, predictive, moderately objective, and user-friendly tools that can help physicians assess prognosis and guide treatment decisions. Future studies should further explore the significance of these scores through prospective research and evaluate how treatment decisions guided by these scores influence patient survival rates.

## Data Availability

The datasets generated and/or analyzed during the current study are not publicly available due to patient confidentiality requirements but are available from the corresponding author on reasonable request.

## Abbreviations

BM: Brain metastasis
UICC: Union for International Cancer Control
KPS: Karnofsky performance status index
GPA: Graded prognostic assessment
molGPA: molecular GPA
WBRT: Whole brain radiotherapy
OS: Overall survival
IC: Intracranial tumor control
IPFS: Intracranial progression-free survival
RT: Radiotherapy
ECM: Extracranial metastases
HR: Hazard ratio
IQR: Interquartile range
